# Hell and High Water: Diminished Septic System Performance in Coastal Regions Due to Climate Change

**DOI:** 10.1371/journal.pone.0162104

**Published:** 2016-09-01

**Authors:** Jennifer A. Cooper, George W. Loomis, Jose A. Amador

**Affiliations:** 1 Laboratory of Soil Ecology and Microbiology, University of Rhode Island, Kingston, Rhode Island, United States of America; 2 New England Onsite Wastewater Training Center, University of Rhode Island, Kingston, Rhode Island, United States of America; Natural Environment Research Council, UNITED KINGDOM

## Abstract

Climate change may affect the ability of soil-based onsite wastewater treatment systems (OWTS) to treat wastewater in coastal regions of the Northeastern United States. Higher temperatures and water tables can affect treatment by reducing the volume of unsaturated soil and oxygen available for treatment, which may result in greater transport of pathogens, nutrients, and biochemical oxygen demand (BOD_5_) to groundwater, jeopardizing public and aquatic ecosystem health. The soil treatment area (STA) of an OWTS removes contaminants as wastewater percolates through the soil. Conventional STAs receive wastewater from the septic tank, with infiltration occurring deeper in the soil profile. In contrast, shallow narrow STAs receive pre-treated wastewater that infiltrates higher in the soil profile, which may make them more resilient to climate change. We used intact soil mesocosms to quantify the water quality functions of a conventional and two types of shallow narrow STAs under present climate (PC; 20°C) and climate change (CC; 25°C, 30 cm elevation in water table). Significantly greater removal of BOD_5_ was observed under CC for all STA types. Phosphorus removal decreased significantly from 75% (PC) to 66% (CC) in the conventional STA, and from 100% to 71–72% in shallow narrow STAs. No fecal coliform bacteria (FCB) were released under PC, whereas up to 17 and 20 CFU 100 mL^-1^ were released in conventional and shallow narrow STAs, respectively, under CC. Total N removal increased from 14% (PC) to 19% (CC) in the conventional STA, but decreased in shallow narrow STAs, from 6–7% to less than 3.0%. Differences in removal of FCB and total N were not significant. Leaching of N in excess of inputs was also observed in shallow narrow STAs under CC. Our results indicate that climate change can affect contaminant removal from wastewater, with effects dependent on the contaminant and STA type.

## Introduction

The soil treatment area (STA; also known as a drainfield or leachfield) of an onsite wastewater treatment system (OWTS) is an important component for removal of contaminants from wastewater. Treatment takes place as wastewater percolates through the unsaturated portion of the soil profile, where low moisture and high oxygen (O_2_) levels are conducive to removal of pathogens, and where chemical and microbial processes can reduce the concentration of other contaminants. The extent of treatment in the STA depends on soil texture, residence time, and the volume of unsaturated soil the wastewater passes through, represented by the vertical separation between the infiltrative surface of the STA and the water table [1–4]. Because wastewater renovation relies on hydrologic, microbial and chemical processes, treatment of wastewater in the STA is sensitive to changes in soil moisture and temperature.

Wastewater contains contaminants that affect human and environmental health. Pathogenic organisms (bacteria, viruses, protozoa and nematodes) can cause illness in humans from ingestion or contact with contaminated water [5]. Excessive nitrate concentration in drinking water disrupts O_2_ binding to red blood cells, known as methemoglobinemia [6], and may cause breathing difficulties in infants. Inputs of nitrogen (N) and phosphorus (P) from OWTS to aquatic ecosystems contribute to eutrophication [7] in marine and fresh waters, respectively. Release of biodegradable organic carbon, as biochemical oxygen demand (BOD_5_), promotes microbial consumption of available O_2_, resulting in hypoxia and death of aquatic organisms [8].

Climate change, through the combined effects of temperature and sea level rise, is expected to affect contaminant removal in the STA in the Northeastern United States. Sea level rise will reduce the volume of unsaturated soil available for wastewater treatment in coastal areas. The sea level in the Northeastern U.S. is projected to rise 90–120 cm by 2100 [9], resulting in higher water tables in coastal regions as denser saltwater displaces lighter freshwater. The effect of sea level rise on water table elevation will diminish with distance from the coast, affect areas with shallow water tables, and depend on the hydrology of the region. A USGS study of Cape Cod found median water table elevation to increase 19–63 cm for modeled sea level elevations between 60 and 180 cm, with 30 cm or greater elevations occurring within approximately 10 km of the coast [10]. Furthermore, precipitation events are expected to increase in number and severity over the same time period [9]. The effects of greater evapotranspiration experienced during warmer temperature [11] is likely to mitigate and often exceed increased precipitation rates on an annual basis, however, at shorter temporal and smaller spatial scales, precipitation may be very large when there is little evapotranspiration. Higher groundwater tables will result in a wetter conditions that enhance the survival and transport of bacterial and viral pathogens [12,13]. Wetter soils may also result in microbial metal reduction, leading to lower P removal capacity and increased mobilization of P retained on soil particles [14]. In contrast, removal of N by microbial reduction to N_2_ may be enhanced by diminished O_2_ diffusion in wetter soils [15]. Finally, decomposition of organic carbon may be hindered or enhanced by increased soil moisture [16], which will affect BOD_5_ removal. Because 40% of the U.S. population resides in coastal communities [17], sea level rise will likely impact coastal communities that rely on OWTS for wastewater renovation.

Elevated temperatures due to climate change may also affect contaminant removal in the STA. Atmospheric temperature is expected to increase 3–5 °C in the next 100 years in the Northeastern U.S, warming the soil profile [9], and warmer conditions have been shown to increase bacterial and viral pathogen mortality [18,19]. Microbial activity increases with warmer temperatures, which may enhance removal of BOD_5,_ however, lower levels of BOD_5_ may limit heterotrophic processes such as N removal by denitrification. Higher temperatures will also reduce O_2_ solubility and increase microbial O_2_ consumption, resulting in less O_2_ available for aerobic treatment processes. In addition, this reduction in available O_2_ can lead to low redox conditions, resulting in metal reduction and a diminished P removal capacity of soil.

In a conventional OWTS, solids are removed from wastewater by sedimentation in the septic tank, and septic tank effluent (STE) is dispersed to the STA for final treatment. The STA in a conventional OWTS is located deep in the soil profile–generally in the C horizon–where infiltration of STE into coarser textured soil with larger pores reduces the likelihood of hydraulic failure due to clogging. Shallow narrow drainfields, an alternative type of STA used with advanced wastewater treatment systems (designed to reduce waste strength, not designed to remove N), may be more resilient to climate change effects than conventional STAs. A shallow narrow STA receives effluent that has undergone secondary treatment in an advanced treatment component, resulting in higher dissolved oxygen levels, and reduced levels of BOD_5_ and particulates in effluent prior to STA dispersal. The secondary treatment lowers the probability of hydraulic failure due to clogging of soil pore spaces, and allows the infiltrative surface to be placed higher in the soil profile than in a conventional STA. Shallower dosing affords a greater volume of unsaturated soil for treatment, and may provide better oxygenation, as well as enhanced filtration of wastewater through finer soil particles in the upper portion of the soil profile. In addition, shallow narrow STA designs incorporate frequent timed-dosing of small volumes of wastewater, preventing prolonged periods of soil saturation, which are common in a conventional STA. Together, these factors may make shallow narrow STAs more resilient to climate change than conventional STAs.

In a previous study we compared the water quality functions of conventional and shallow narrow STAs [20]. We observed complete removal of fecal coliform bacteria (FCB), bacteriophage (a human virus surrogate) and total P, and near complete removal of BOD_5_ in conventional and shallow narrow STAs [20]. Although limited, removal of total N was higher in the conventional STA [20]. In the present study we tested the hypothesis that climate change (higher shallower water table and increased temperature) would diminish removal of FCB, viral surrogates, and total P in conventional and shallow narrow STAs, whereas removal of BOD_5_ and total N would be marginally improved. We expected the shallow narrow STAs to have comparatively better contaminant removal than conventional STAs because the former have a larger volume of soil for treatment. We evaluated these hypotheses in a laboratory experiment using triplicate intact soil mesocosms representing a conventional STA and two types of shallow narrow STAs. We compared the water quality functions of the STAs under present climate (PC) (20°C; vertical separation distance representative of regulatory values), and moderate 100-year predicted climate change (CC) (25°C; vertical separation distance reduced by 30 cm by raising the water table elevation) conditions. These conditions are generally representative of the expected climate changes in the glaciated Northeastern U.S.

## Materials and Methods

### Description of mesocosms

**Fig 1:**
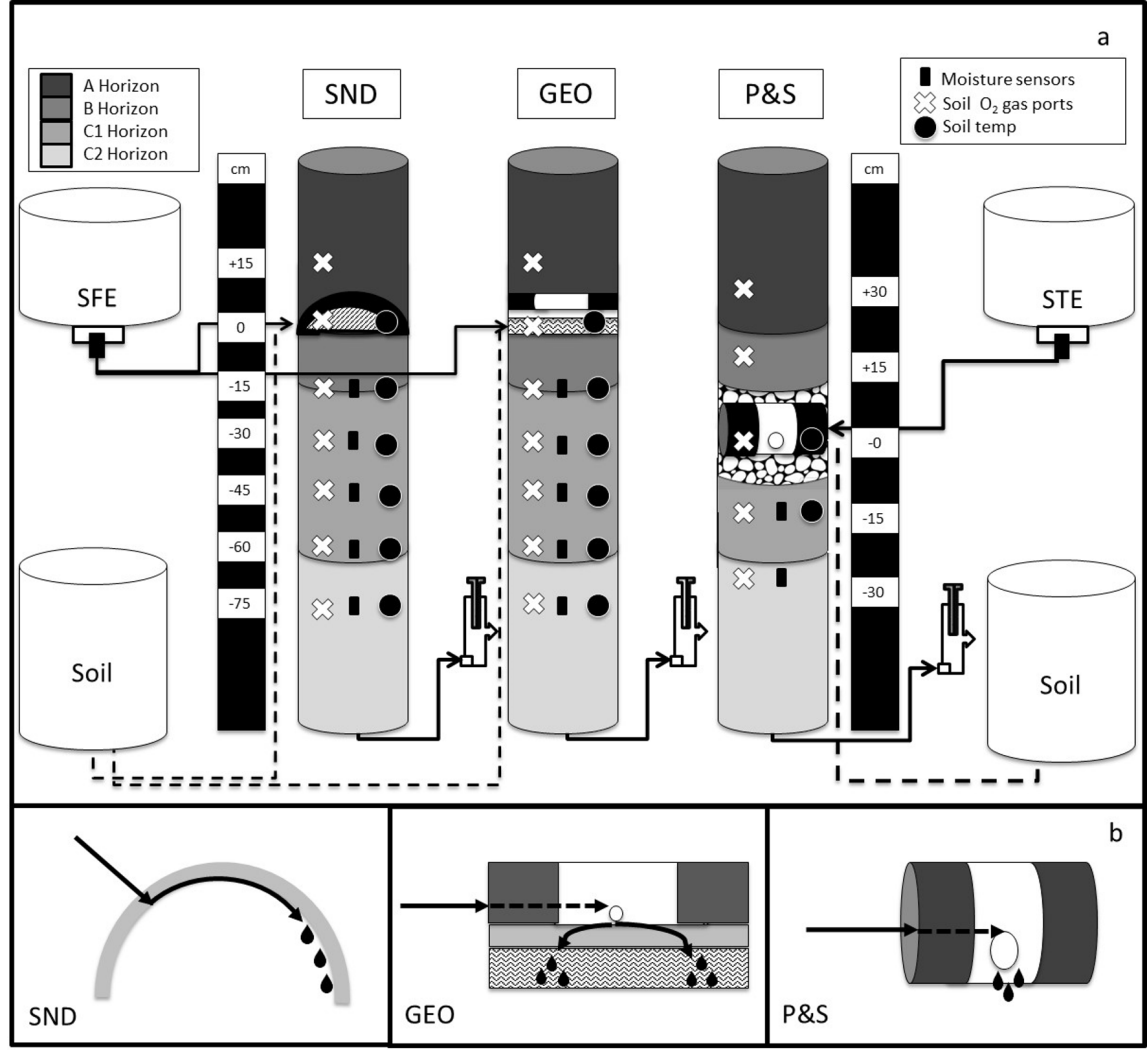
**(A) Schematic diagram of soil mesocosms representing a shallow narrow drainfield (SND), GeoMat^®^ (GEO), and pipe and stone (P&S) soil treatment areas (STAs).** The wastewater input to SND and GEO was sand filter effluent (SFE), whereas the P&S received septic tank effluent (STE). The approximate location of soil horizons, ports for gas sampling, and moisture and temperature probes are indicated. Water exits the mesocosms through a hanging water column device used to adjust the height of the water table. The atmosphere in the infiltrative area is connected to a 30-cm soil column. **(B) Detailed schematic diagram of the SND, GEO and P&S delivery devices.** Diagrams are not to scale. Heating cables were wrapped around mesocosms, covered with insulation, and connected to a digital thermostat to control soil temperature.

Intact soil cores were collected in PVC pipes (152-cm tall x 15-cm-diam.), and excavated in October 2012 from a 5-m long trench containing Bridgehampton silt loam soil (Coarse-silty, mixed, active, mesic Typic Dystrudepts) in Kingston, Rhode Island, USA. Bottom edges of PVC pipes were sanded until sharp to minimize soil compaction and gradual pressure was applied to the top with an excavator until desired core depth was achieved. Intact cores were transported to the laboratory (< 1 mile) in a flat-bed truck and engineered to represent one of three drainfield types in the laboratory:(i) conventional pipe and stone (P&S), (ii) shallow narrow drainfield (SND), and (iii) Geomat® (GEO), a SND variation (Fig 1), in triplicate. The infiltrative area was established at 20 cm below the ground surface for SND (Fig 1), at 25 cm for GEO, and at 84 cm for P&S. Briefly, dosers were designed to replicate technology designs with sheet flow inside an upside down, halved 10-cm-diam. PVC dome for SND, diffused flow from a 2.5-cm-diam. PVC pipe through fused plastic filament mesh and geotextile filter fabric for GEO, and massive flow from a 10-cm-diam. PVC pipe placed between layers of stone for P&S. Mixed A and B horizon topsoil covered dosers to previous soil surface level as per in-field practices (Fig 1). A detailed description of the experimental design, sampling, and analytical methods can be found in [20].

### Moisture

The separation distance from the water table was controlled using a hanging water column (Fig 1) and, to represent present climate (PC) conditions, was set at 102 cm below the infiltrative surface for SND and GEO, and at 56 cm for P&S to replicate technology regulations [21,22]. To simulate climate change (CC), the water table was raised 30 cm, resulting in a separation distance of 72 cm for SND and GEO, and 26 cm for P&S. Soil EC-5 moisture probes and Em5b data loggers (Decagon Devices, Pullman, WA) were used to measure soil moisture every 15 minutes at depths of 15, 30, 45, 60 and 75 cm below the infiltrative surface for SND and GEO, and at 15 and 30 cm below the infiltrative surface for P&S (Fig 1).

### Temperature

The mesocosms were maintained at 20.0 ± 0.7°C under PC conditions, and the temperature increased to 25.0 ± 0.7°C to simulate CC. This was accomplished by covering the outside of the mesocosms with heavy-duty aluminum foil (to increase heat diffusion), wrapping 115V heating cables (Hydrokable, Sacramento, CA) around the mesocosms, and wrapping reflective double bubble foil insulation material around the mesocosms. A thermostat (NEMA 4X, Aqua Logic, Inc., San Diego, CA) was used to regulate the temperature, and soil temperature was measured using iButton sensors (DS1921G, Maxim Integrated, San Jose, CA) buried 5 cm below the soil surface. Ceramic-tipped probes (YSI, Yellow Springs, OH) were used to measure soil temperature at depths of 0, 15, 30, 45, 60 and 75 cm below the infiltrative surface for SND and GEO, and at 0 and 15 cm below the infiltrative surface for P&S (Fig 1).

### Wastewater dosing and characteristics

The P&S was dosed with 200 mL of septic tank effluent (STE) every 12 h over 1.5 h, corresponding to 400 mL d^-1^ (22.6 L m^-2^ d^-1^). The SND and GEO received wastewater that had passed through a single-pass sand filter (SFE). They were dosed with 22.5 mL SFE every 30 min over 15 min, corresponding to 2000 mL d^-1^ (113 L m^-2^ d^-1^). Dosing rates were based on regulations governing OWTS design in the state of Rhode Island [21,22]. Septic tank effluent and SFE were collected weekly from the same treatment train at a residence in South Kingstown, RI, USA. Permission was provided by the homeowners to access and sample their septic system for the duration of this study. Wastewater was stored in sealed plastic containers at 4°C in the dark after collection before dispensing small volumes to chilled plastic holding containers maintained at 4–16°C for dosing. The characteristics of wastewater inputs (Table 1) are within the range observed by others [23–25].

**Table 1:** Characteristics of septic tank effluent (STE) and sand filter effluent (SFE) used in our study under present climate (n = 8 samples) and climate change (n = 11 samples) scenarios.

Analyte	STE		SFE	
	Present	Climate	Present	Climate
	climate	change	climate	change
pH	6.3 ± 0.2	6.5 ± 0.2	3.9 ± 0.9	4.6 ± 0.5
Dissolved O_2_	0.0 ± 0.0	0.0 ± 0.0	2.4 ± 0.6	3.2 ± 1.3
BOD_5_	219 ± 61	140 ± 79	11 ± 7.3	6.1 ± 8.6
Electrical conductivity	786 ± 47	620 ± 146	615 ± 85	422 ± 120
Fecal coliform bacteria	1.3 × 10^6^	1.1 × 10^5^	4.2 × 10^3^	1.6 × 10^1^
	± 1.7 × 10^6^	± 1.2 × 10^5^	± 6.7 ×10^3^	± 2.8 × 10^1^
Total N	67 ± 8.0	52 ± 15	58 ± 8.0	44 ± 11
NH_4_-N	50 ± 7.0	36 ± 15	10 ± 4.8	5.7 ± 2.9
NO_3_-N	0.02 ± 0.04	0.02 ± 0.02	40 ± 8.0	24 ± 8.7
Total P	9.1 ± 0.6	7.4 ± 2.1	7.8 ± 1.2	6.3 ± 1.6
PO_4_-P	6.9 ± 0.4	5.70 ± 1.7	50 ± 0.4	4.7 ±1.2
SO_4_-S	9.3 ± 1.3	8.5 ± 2.4	15 ± 2.8	13 ±3.9
Field collection temperature	20 ± 2.0	12 ± 6.1	20 ± 2.2	11 ± 6.8

Values are means ± standard deviation. All units are mg L^-1^ except for pH, electrical conductivity (μS), fecal coliform bacteria (CFU 100 mL^-1^), and collection temperature (°C).

### Analyses

Output water was collected at the bottom of the mesocosms under both climate conditions, in N_2_-purged, autoclaved 1-L Nalgene bottles fitted with an airlock, and the water was analyzed for pH, dissolved O_2_, BOD_5_, electrical conductivity, FCB, total N, ammonium, nitrate, total P, phosphate, and sulfate, as described in [20]. Samples for Al, Fe and Mn were acidified to pH<2 with HCl, and analyzed at the Brown University Environmental Chemistry Facility with a JY2000 Ultrace ICP Atomic Emission Spectrometer (Horiba, Kyoto, Japan) equipped with a JY AS 421 autosampler and 2400 g mm^-1^ holographic grating. Details of MS2 viral measurement of concentration can be found in S1 File—Supplemental Methods.

### Timeline

Mesocosms were prepared by initially filling with tap water from the bottom, and dosed with tap water at steady flow rates for 75 days. They were allowed to drain by gravity for two days before introduction of wastewater. The mesocosms received wastewater for 24 months prior to this experiment. The data representing PC in this study was collected for four months (July–Oct., 2014) prior to implementation of CC conditions. Climate change data was collected after the STAs had equilibrated, approximately four months after the change in climate conditions (Feb.–July, 2015). We estimated the time required for equilibration of STAs based on the time for recovery of water quality functions following environmental disturbances reported in [26], as well as stabilization of variation in water quality functions following elevation of water table and temperature in our experiment.

### Damköhler number

We evaluated N removal in the STA using the Damköhler number (*D*_*a*_) [27], which compares the timescales of transport and reaction rate. Values *D*_*a*_ < 1 indicate that elevated rates of transport limit denitrification, and *D*_*a*_ > 1 indicate that reactant (e.g. NO_3_^-^) consumption limits denitrification [28]. This approach has been employed successfully by others to identify the extent to which transport and biochemical reactions control removal of N in groundwater and riparian zones [28,29]. The Damköhler number was calculated using the equation:

$$D_{a}=kC_{0}^{n-1}\tau$$ (1)

where *D*_*a*_ = Damköhler number (unitless), *k* = reaction constant, zero-order (mg L^-1^ h^-1^), *C*_*0*_ = initial concentration of nitrate (mg L^-1^), n = reaction order (zero order), τ = mean residence time = *L/v* (h), where *L* = distance between sample ports (cm) and *v* = velocity (cm h^-1^) [27].

### Statistics

A non-parametric two-way ANOVA was used to evaluate differences in removal of BOD_5_, FCB, total P, and total N as a function of STA type and climate conditions using untransformed data, except for total P, which was transformed using a 1/(n) transform. Means separation was accomplished using the Holm-Sidak method. A t-test was used to evaluate differences in input wastewater characteristics for STE and SFE between climate conditions. All statistical tests were performed on averaged replicate data by sampling date collected over four months, and evaluated at p ≤ 0.05.

## Results and Discussion

### Increased moisture and lower O_2_ under climate change

**Fig 2:**
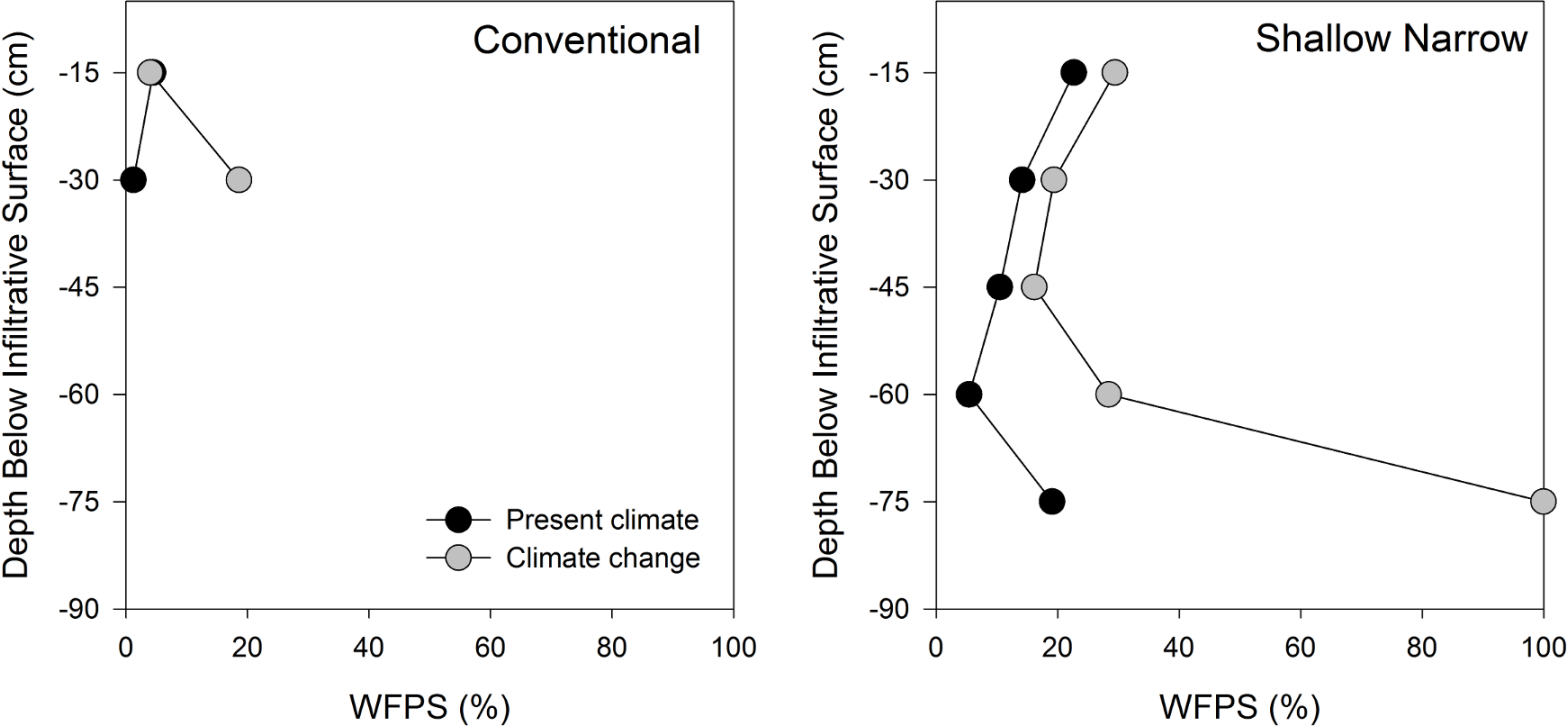
Water-filled pore space (WFPS) in conventional (P&S) and shallow narrow (SND and GEO) soil treatment areas under present climate and climate change. Values represent the average WFPS over 24 h at each depth.

**Fig 3:**
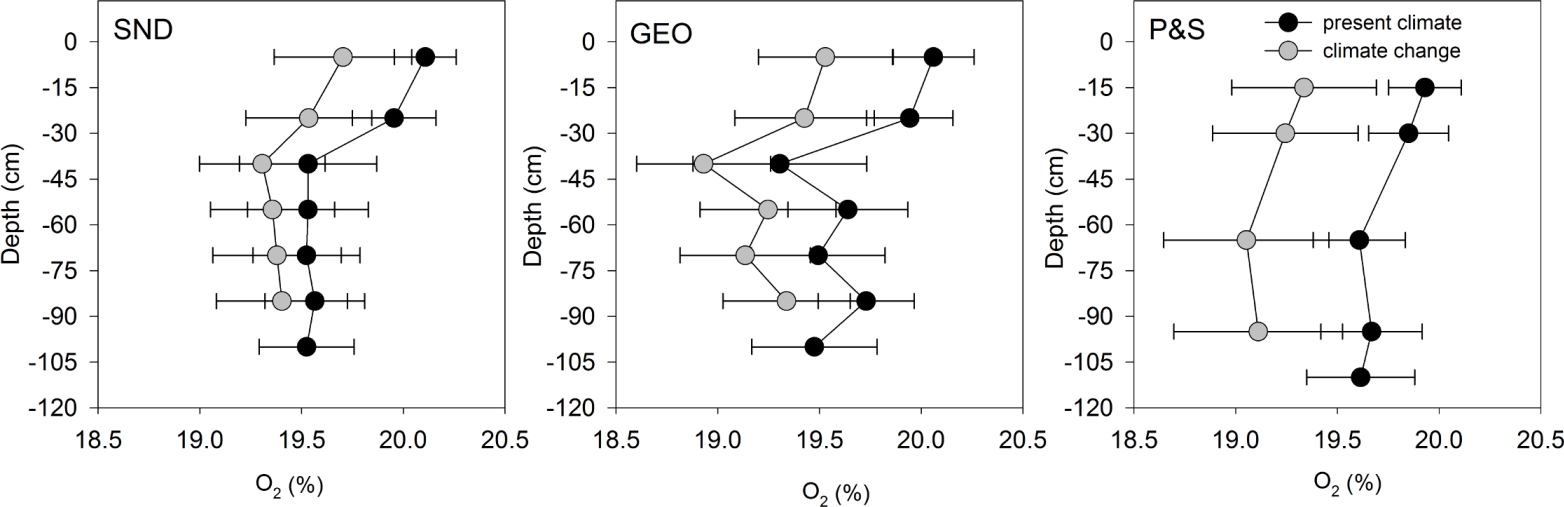
Soil pore O_2_ concentration under present climate and climate change for shallow narrow (SND and GEO) and conventional soil treatment areas. Values are means (n = 3); error bars represent one standard deviation for a single mesocosm over four months.

Climate change was expected to result in wetter soils with lower O_2_ relative to PC in all three STAs. Water-filled pore space (WFPS) increased under CC for conventional and shallow narrow STAs at all depths (Fig 2). Values of WFPS for P&S ranged from 3%-11% under PC and increased to 10%-47% under CC, whereas WFPS increased from 5%-23% to 16%-29% in the shallow narrow STAs under CC. The concentration of O_2_ in soil pores was lower under CC relative to PC at all depths (Fig 3). Less O_2_ and higher WFPS in the STA can have a number of consequences for contaminant removal processes, discussed below.

### More BOD_5_ removal under climate change

**Fig 4:**
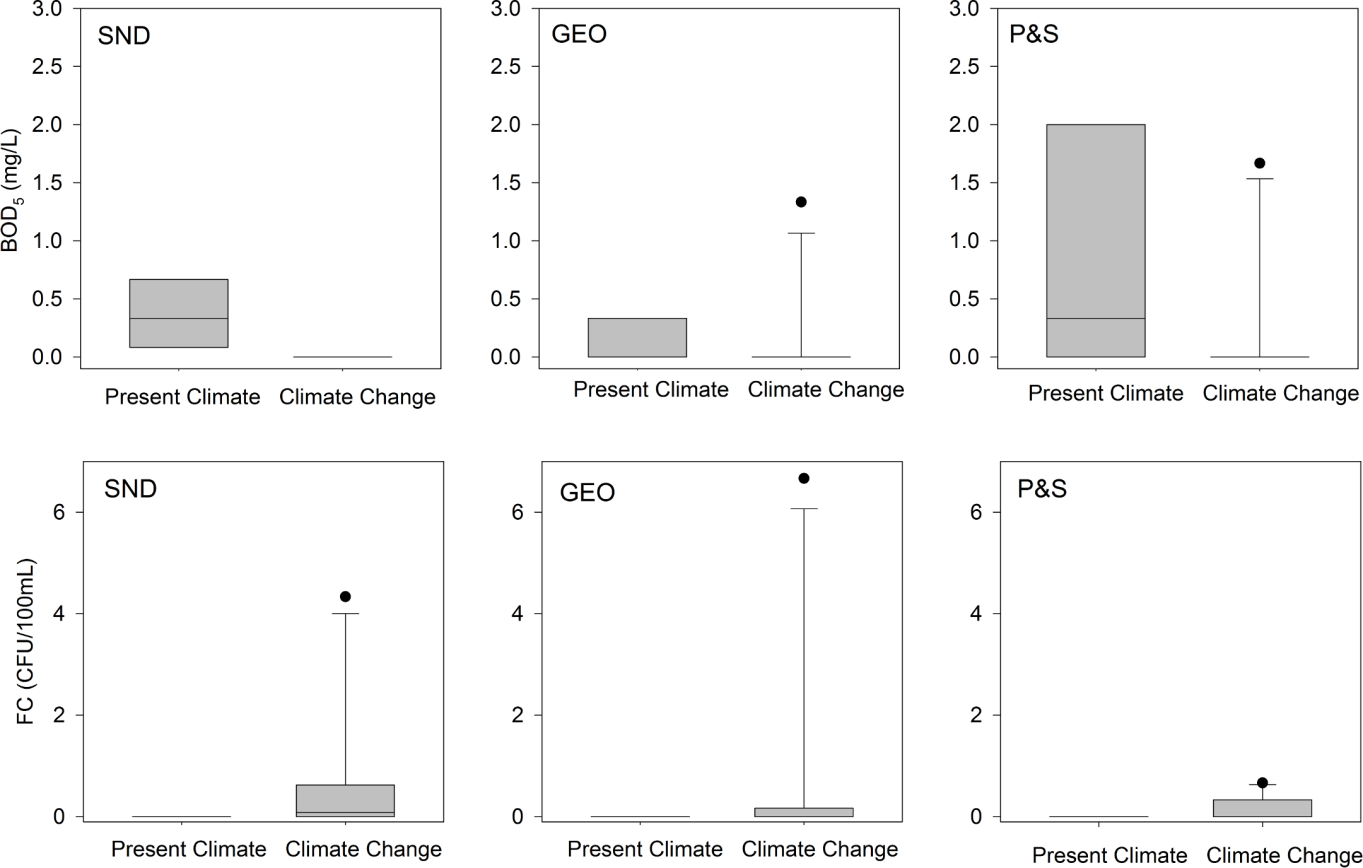
**Concentration of biochemical oxygen demand (BOD_5_) (top) and fecal coliform bacteria (FCB) (bottom) in output water under present climate and climate change for shallow narrow (SND and GEO) and conventional (P&S) soil treatment areas.** Values are means (n = 3) at each sampling date. Boxes represent the median and interquartile range, whiskers represent the 10^th^ and 90^th^ percentiles, and dots represent values outside the 10^th^ and 90^th^ percentiles for all sample dates.

Climate change resulted in a decrease in the median concentration of BOD_5_ in output water from 0.3 to 0.0 mg L^-1^ for P&S and SND, and remained at 0.0 mg L^-1^ for GEO (Fig 4). The concentration of BOD_5_ in output water was significantly different between climate conditions (p = 0.011), but not among STA types (p = 0.699) (Fig 4). Pairwise comparisons of means between STA types were not significant under either climate condition. However, there was significantly less BOD_5_ present in input wastewater to P&S. Variability in the concentration of BOD_5_ in output water was higher for GEO and P&S under CC in comparison to PC, whereas variability in output BOD_5_ concentration in SND was similar between climate conditions.

#### Less BOD_5_ can limit heterotrophic processes

Our results generally support the hypothesis that BOD_5_ removal may increase under CC. Soil microbial communities are carbon limited [30], and BOD_5_ is expected to be well removed in the STA. Decomposition of organic carbon in soil is dependent on soil moisture content and temperature [15,16]. Because both soil moisture and temperature increased with CC, we cannot ascertain the contribution of each variable to increased BOD_5_ removal. Greater removal of BOD_5_ (Fig 4) under CC in SND and GEO may have limited heterotrophic processes in the STA, such as denitrification, as discussed below.

### Release of FCB increased under climate change

No FCB were detected in output water under PC (Fig 4). In contrast, FCB was detected in output water from all three STA types under CC, with maximum concentrations of 17, 6, and 20 CFU 100 mL^-1^ for P&S, SND and GEO, respectively. Median output water concentrations were 0.0 CFU 100 mL^-1^ for P&S and GEO, and 0.1 CFU 100 mL^-1^ for SND. Differences were not statistically significant between climate conditions (p = 0.106) or among STA types (p = 0.696). The presence of FCB in output water was more variable under CC for all STA types, with greater variability observed in SND and GEO (Fig 4).

#### Wetter soil likely reduced microbial attachment

Unsaturated conditions favor FCB removal in the STA [3,31] by increasing the opportunity for attachment to soil particles. Increased moisture content likely reduced bacterial attachment to soil, resulting in more FCB in output water under CC. Greater bacterial survival has also been observed in wetter soils [12]. Growth of FCB may also have taken place under CC, as indicated by higher numbers of FCB in output water from SND and GEO under CC relative to inputs from SFE on two out of 11 sampling events (data not shown). Others [32,33] have observed the survival and propagation of *E*. *coli* in soil. Generally, bacterial pathogens in soil experience increased mortality with increased temperatures [18,34]. However, our results suggest that the combination of warmer and wetter soils may have enhanced the transport, survival and/or growth of FCB in all STA types.

#### Temperature likely less important than moisture for FCB removal

A study modeling *E*. *coli* transport in the STA at 20°C and 23°C found greater attenuation at the higher temperature, but predicted lower *E*. *coli* removal under simulated increases in rainfall leading to wetter soils [34]. These two effects were not coupled in the model; since we observed lower FCB attenuation in our experiment, this would suggest that the degree of soil moisture plays a larger role in bacterial removal than temperature.

Our results suggest the possibility of greater presence of FCB–and thus pathogenic bacteria—in output water, particularly if bacterial growth takes place under CC. Many pathogenic microorganisms require relatively small doses to cause illness in humans. For example, *E*. *coli* O157:H7, which produces shiga toxin and can cause kidney failure and death, requires fewer than 10 cells to cause illness [35], while an infected human will release 10^5^−10^8^ cells in feces [36].

### Virus removal unlikely to be impacted by climate change

We determined the effects of CC on the fate and transport of viruses using MS2 bacteriophage, a surrogate for human viruses [37]. MS2 was not detected in output water from any STA type under PC or CC (data not shown). Greater virus transport and survival has been observed in wetter soils [13]; however, virus inactivation generally increases with increased temperature [19].

#### Acidic soils may be important for viral removal

The absence of differences in virus removal between climate conditions and among STA types suggests a common mechanism for viral removal and inactivation under all of these conditions. Viral particles develop a positive charge at pH values below their isoelectric point (pI). The pI of most bacteriophage and animal viruses is < 7.0 [38], and the pH of the soil in the STAs was < 3.5 [20], suggesting that viruses are likely retained on the negatively-charged soil surfaces. This ionic interaction is probably more important than the effects of temperature or soil moisture on the fate and transport of viruses in these STA types.

The absence of viruses in output water regardless of climate conditions has positive consequences for public health. Enteric viruses can cause illness in humans from ingestion of a single viral particle [39], and the feces of a human infected with rotavirus contains up to 10^7^ viral particles [36]. Our results are encouraging for virus removal in the STA, however, soils with higher pH and greater buffering capacity may not have a comparable virus removal capacity.

### Effects of climate change on N removal dependent on STA type

**Fig 5:**
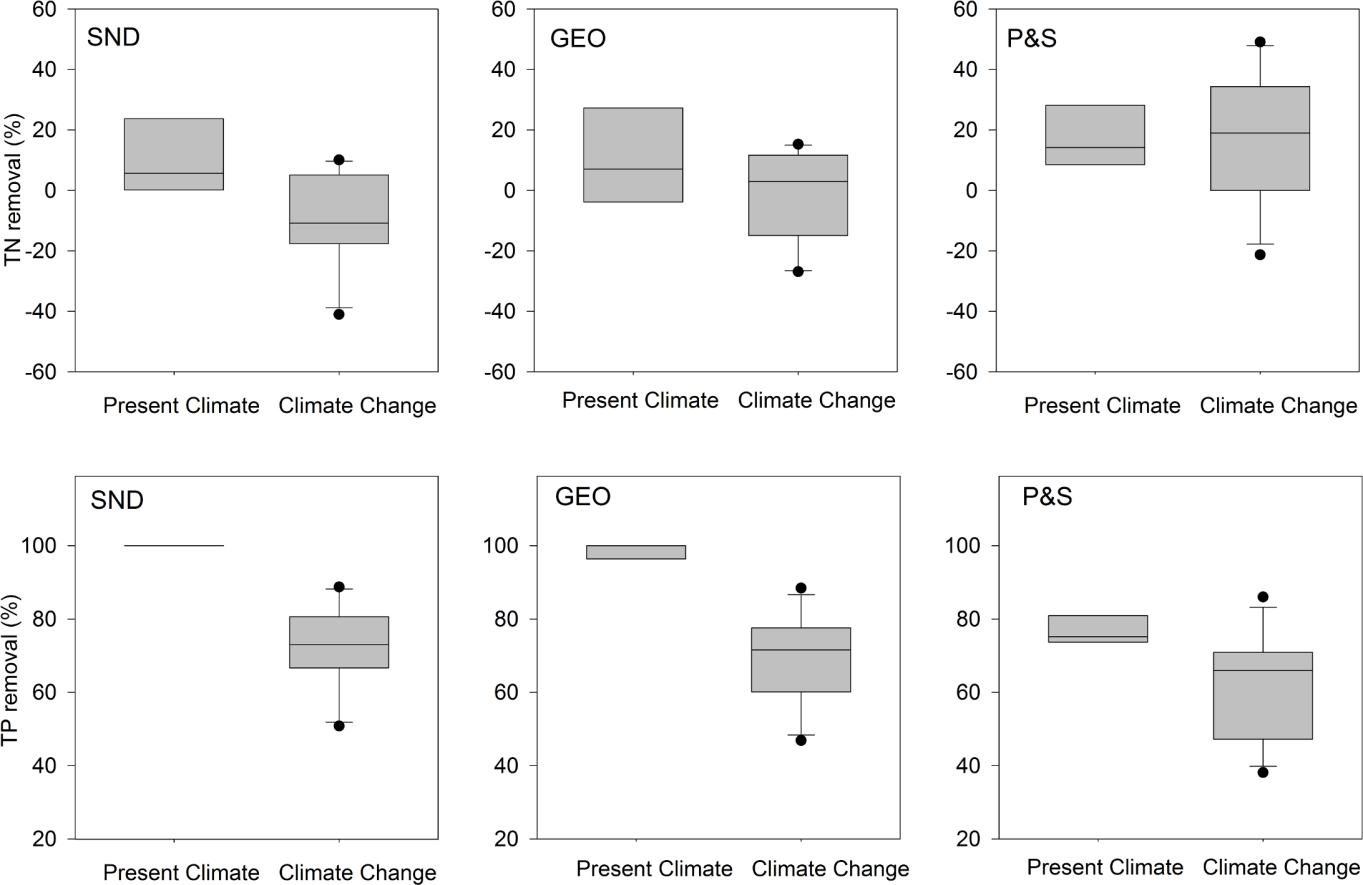
**Total nitrogen (top) and total phosphorus (bottom) removal under present climate and climate change for shallow narrow (SND and GEO) and conventional (P&S) soil treatment areas.** Values are averages of three replicates by sampling date. Values are means (n = 3) at each sampling date. Boxes represent the median and interquartile range, whiskers represent the 10^th^ and 90^th^ percentiles, and dots represent values outside the 10^th^ and 90^th^ percentiles for all sample dates.

The median total N removal was lower under CC for SND and GEO (Fig 5), decreasing from 6% to -11% in SND and from 7% to 3% in GEO, resulting in a net increase in total N concentration in output water relative to PC conditions. In contrast, the median total N removal for P&S increased, from 14% under PC to 19%, under CC (Fig 5). The differences in total N removal between climate conditions were not significant (p = 0.171), although differences in removal among STA types were (p = 0.008). Pairwise comparisons of means between STA types indicated that P&S was significantly different from SND or GEO; however, there was no significant difference in N removal between SND and GEO. Total N removal was more variable under CC for all STA types, which likely contributed to the absence of a statistically significant effect of climate conditions. There were more events of no net change in N concentration or net increase in total N concentration in output water under CC (data not shown). Under PC, 25% (SND) and 100% (GEO and P&S) of the observations reflected net removal of total N. However, 27% (SND) and only 63% (GEO and P&S) of CC observations resulted in net removal of total N. Although the frequency of total N leaching events in SND were similar between climate conditions, the concentration N in the output water was higher under CC.

Our results suggest that CC may increase inputs of N to ground water from shallow narrow STAs. Higher inputs of N to groundwater under CC increases the probability of affecting ecosystem and public health. Eutrophication from excessive inputs of N to saline water bodies may lead to hypoxia and anoxia when microorganisms decompose plant material after death, killing fish and other aerobic organisms. High levels of NO_3_^-^ in ground water may also increase the risk of methemoglobinemia in infants.

#### Heterotrophic N removal limited under climate change

Lower availability of organic carbon, measured as BOD_5_ (Fig 4), likely contributed to lower total N removal in SND and GEO under CC. Heterotrophic denitrification is considered to be the primary mechanism for N removal in the STA [40–42], and requires organic carbon as an electron donor to produce N_2_ and N_2_O from nitrate (NO_3_^-^). The shallow narrow STAs receive SFE, which has a low initial concentration of BOD_5_ as a result of passage through an aerobic sand filter (Table 1). Greater removal of BOD_5_ (Fig 4) under CC in SND and GEO may have limited heterotrophic denitrification in these STAs, particularly if BOD_5_ removal takes place closer to the infiltrative surface. In contrast, the P&S drainfield receives STE, which has a higher initial concentration of BOD_5_ (Table 1), and organic carbon availability may not limit total N removal (Fig 5).

The leaching of total N in excess of inputs may be due to an increase in the frequency of N cycling. In a previous study [43], we presented evidence of internal recycling of N through uptake and re-mineralization of microbial biomass N. Establishment of climate change conditions, and more frequent incidents of water table perching [43] in SND and GEO, may have caused the rate of internal N recycling to increase, increasing the probability that sampling occurred when net leaching of N to output water took place.

Microbial processes other than heterotrophic denitrification can contribute to N removal in the STA, including N_2_O production from nitrification [44], N_2_ production from anaerobic ammonia oxidation [45], and N_2_ production from autotrophic denitrification [46,47]. We have shown evidence for the occurrence of these processes in the STA [43]. Lower available O_2_ (Fig 3) due to warmer and wetter conditions under CC would be expected to favor N removal by both autotrophic and heterotrophic processes in all three STAs. However CC may have impacted heterotrophic N removal negatively due to organic carbon limitations in shallow narrow STAs.

#### Rapid movement of wastewater in STA may limit N removal

**Fig 6:**
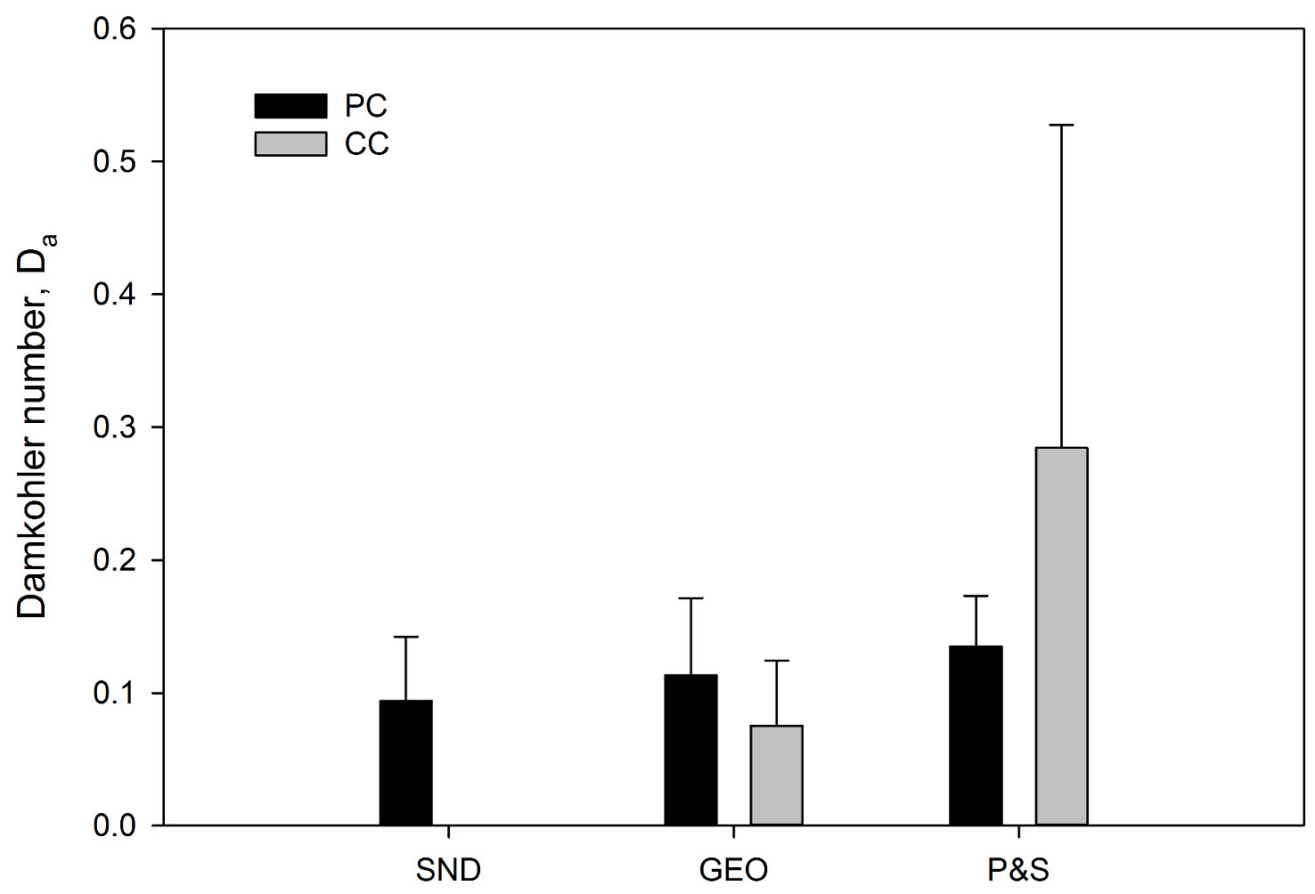
Damköhler number (*D*_*a*_) values under present climate (PC) and climate change (CC) for N removal in shallow narrow (SND and GEO) and conventional (P&S) soil treatment areas. Values are means of the range of possible values and error bars represent one standard deviation of the mean.

We evaluated N removal in the STA using the Damköhler number (*D*_*a*_) [27], which compares the timescales of transport and reaction rate. Our analysis shows that rapid movement of water through the STA limits N removal (Fig 6), as indicated by values of *D*_*a*_ < 1 for all STA types (S1 Table). The residence time of the wastewater is 3–4 times greater in P&S than SND and GEO. Furthermore, the value of *D*_*a*_ is lower for SND and GEO, and higher for P&S under CC relative to PC, reflecting differences in the reaction rates between climate conditions, since we assumed the velocity of water remains the same under both climate regimes. Our results suggest that the movement of water through the STA may be too rapid for substantial denitrification to take place, regardless of STA type and climate conditions (S1 Table). Improvement of total N removal may be achieved through slower water movement, which would allow for higher rates of NO_3_^-^ consumption.

#### Models of N removal need more parameterization

In a model simulation of N removal in our STA mesocosms, Morales et al. [48] predicted increased N removal at 23°C (in comparison to 20°C) under PC depth to the water table, and higher N removal as the water table was elevated. The poor agreement between the modeled simulation and experimental results suggests that additional parameters affected by CC, such as higher consumption of organic C, need to be incorporated in the model.

### Phosphorus removal diminished under climate change

Median total P removal under PC was close to 100% for SND and GEO, and declined to 71% for SND and 72% for GEO under CC (Fig 5). Median total P removal in P&S also declined, from 75% under PC to 66% under CC. The differences in total P removal between climate conditions were significant (p = <0.001), as were differences in removal among STA types (p = 0.004). Pairwise comparisons of means between STA types indicated that P&S was significantly different from SND or GEO; however, SND and GEO did not have different removal. As was the case for removal of other wastewater constituents, we observed higher variability in total P removal under CC than under PC in all STA types (Fig 5).

#### Reduction of metal-P complexes mobilize P

Our results support the hypothesis that total P removal may diminish under CC for all STA types. The mechanism for this effect may involve lower availability of O_2_ in the STA under climate change. Limited O_2_ availability likely lead to microbial reduction and increased solubility of redox-active metals (Fe and Mn) in soil involved in forming insoluble precipitates with phosphate. Along with formation of precipitates with Al oxides, this is thought to be the primary mechanism for total P removal in the STA [14,20]. Reduction of Fe and Mn increases their solubility, which not only releases phosphate bound to Fe and Mn oxides into the dissolved phase, but also results in a decrease in the number of metal oxide sites available for reaction with–and retention–of phosphorus. Dissolution of Al at the acidic pH found in the STA (< 3.5) may have also contributed to P leaching [20], however, the pH of the output water, and likely STA soil (data not run), was similar under both climate conditions.

#### Abiotic mechanisms appear more important for P retention

**Fig 7:**
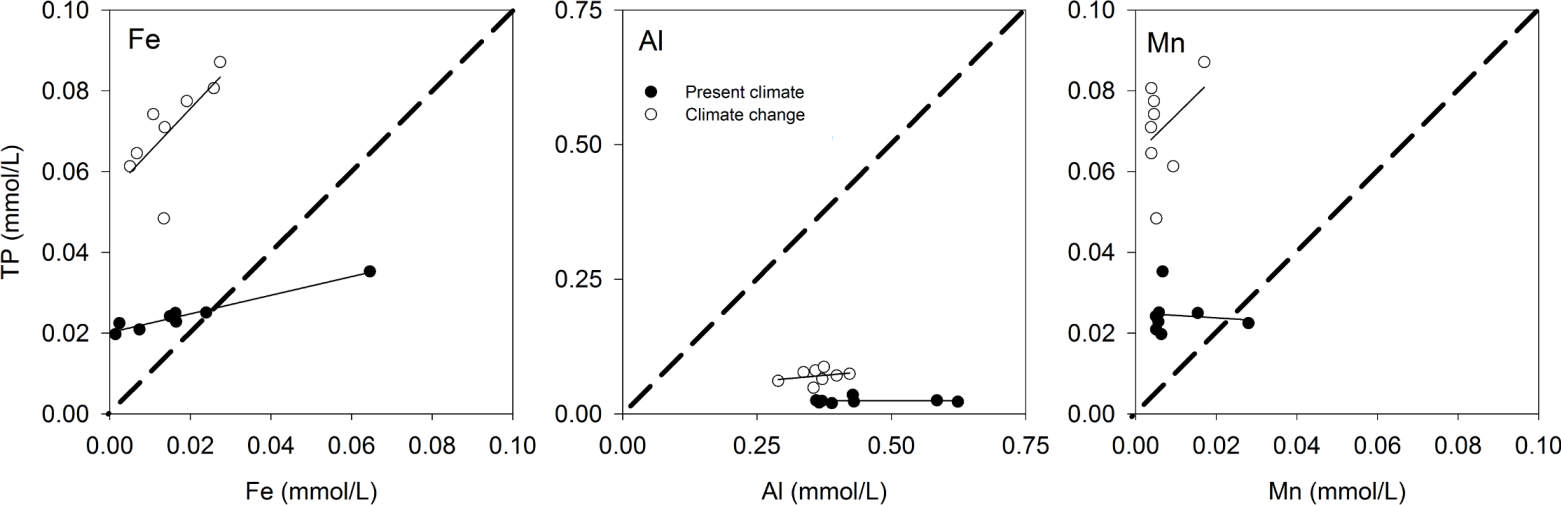
Concentrations of Fe, Al and Mn vs. total P (TP) in output water from all three STA types (n = 8). Dashed line describes the stoichiometry of metal-P complexes.

To differentiate between potential abiotic mechanisms affecting P removal, we plotted the concentration of dissolved Fe, Mn and Al in output water versus the concentration of total P for all three STA types under PC and CCe (Fig 7). The closer the slope of the line of metal concentration vs. P in solution is to 1 (indicating stronger coupling)–which describes the stoichiometry of the metal-P complexes–the more likely it is that P was released from complexes formed with that metal. Under PC, the slope of the line for all three metals is considerably less than 1, indicating that dissolution of metal-P complexes was not responsible for release of total P to output water (Fig 7). In contrast, under CC the slope of the line for Fe and Mn is much closer to 1, suggesting that reduction of these metals became more important for total P release under CC. Because Al and total P in output water were not strongly coupled (Fig 7), it appears that climate change did not influence this mechanism.

Our results suggest that the concentration of total P in output water from the STA may increase under CC. The resulting higher levels of total P in groundwater can eventually lead to increased eutrophication of freshwater bodies. In addition to the detrimental effects of eutrophication on aquatic organisms, it may also lead to public health concerns related to production of trihalomethanes (THM), carcinogenic compounds produced from chlorination of drinking water with a high concentration of dissolved organic carbon [49]. Algal blooms can also result in the production of human toxins that prevent use of surface water for drinking [50,51].

### Whole system evaluation

**Fig 8:**
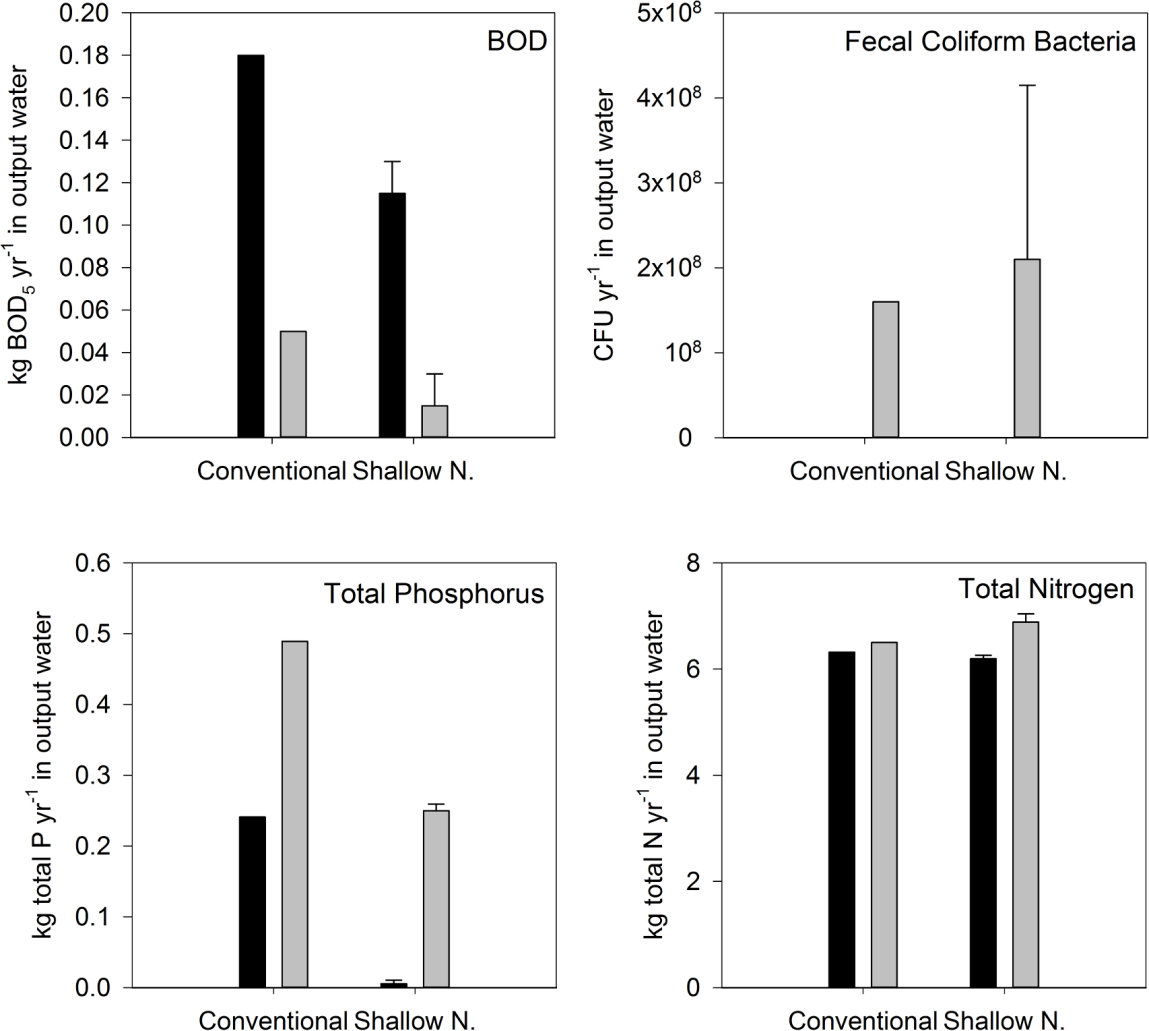
Yearly estimates of mass of BOD_5_, total P and total N, and number of fecal coliform bacteria released to groundwater from treatment trains that include P&S and shallow narrow (SND & GEO) soil treatment areas under present climate (dark bars) and climate change (light bars). Error bars represent difference in removal between SND and GEO.

To compare the performance of the treatment trains that include conventional and shallow narrow STAs under PC and CC, we estimated contaminant removal over the course of a year (Figs 8 and S1). We used mean values for contaminant concentrations in input water (Table 1) and, for treatment trains including SND and GEO, assumed that contaminant removal rates in the sand filter did not change with climate change. At the system scale, more BOD_5_ was released from a treatment train with a conventional STA than from treatment trains with shallow narrow STAs under both climate conditions, likely due to sand filter pre-treatment. A greater number of FCB were present in output water from systems with a shallow narrow STAs than a system with a conventional STA under CC conditions, whereas complete FCB removal was observed under PC in all three systems. A higher mass of total P was released from treatment trains with a conventional STA than from treatment trains with shallow narrow STAs under both climate conditions. A greater mass of total N was present in output water from systems with a conventional STA under PC, however, more total N was present in output water from systems with shallow narrow STAs under CC (Fig 8). Because the shallow narrow systems are not designed to enhance N removal, use of alternative OWTS with advanced N removal components should improve N removal rates regardless of climate conditions.

## Conclusions

Our results indicate that CC can affect contaminant removal, with effects dependent on the contaminant and STA type. Removal of FCB, total P and total N in shallow narrow STAs diminished under CC conditions. In contrast, total N removal in conventional STAs improved. Viral pathogens and BOD_5_ were well removed under PC and CC, suggesting that OWTS were more resilient with respect to these contaminants.

Although conditions in the field may diverge from those in the laboratory, our experiment allowed us to make direct comparisons between PC and CC among different STA types. We recognize that systems installed under field conditions have more performance variability than systems evaluated under laboratory conditions [52]. Warming the entire STA, as opposed to only the near surface under field conditions, enabled us to make direct observations between the two temperature conditions at all depths in the soil profile. While the length of our study was relatively short, the limited duration prevented extreme temporal variation in the STA microbial communities between climate conditions.

The response of abiotic and biotic components in OWTS to differing temperature and moisture conditions may be different. For example, the rate of soil microbial processes is known to double with a 10°C increase in temperature [53]. In contrast, the rate of chemical reactions generally respond in a linear manner to increases in temperature over the range of temperatures experienced by soil [54]. Nevertheless, our results demonstrate the potential effects of climate change on different types of OWTS. This study provides regulators with a starting point for future planning as well as providing an impetus for designing improvements for OWTS technologies.

## Supporting Information

S1 Fig Estimates of field-scale mass loading from septic tank, sand filter and soil-based treatment for an advanced system with a shallow narrow (SND) or GeoMat^®^ (GEO) soil treatment area, and for a conventional system with a pipe and stone (P&S) soil treatment area. Removal values (%) are for the previous step in the treatment train. Units are kg yr^-1^ except for fecal coliform bacteria (FCB), which are CFU yr^-1^.
(PDF)

S1 File Supplemental Methods. Methodology for the propagation, addition and detection of MS2 bacteriophage in the pipe and stone (P&S), shallow narrow (SND) and Geomat® soil treatment areas (STAs).
(PDF)

S1 Table Measured parameters used to calculate the Damköhler Number (*D*_*a*_) under present climate (PC) and climate change (CC) conditions for nitrate removal within shallow narrow (SND/GEO) and conventional (P&S) soil treatment areas. (PDF)
