# Supplementary figures and images for: Hell and High Water: Diminished Septic System Performance in Coastal Regions Due to Climate Change
Source: PLoS One. 2016 Sep 1;11(9):e0162104. doi: 10.1371/journal.pone.0162104 (PMC5008777; doi:10.1371/journal.pone.0162104)

S1 Fig.

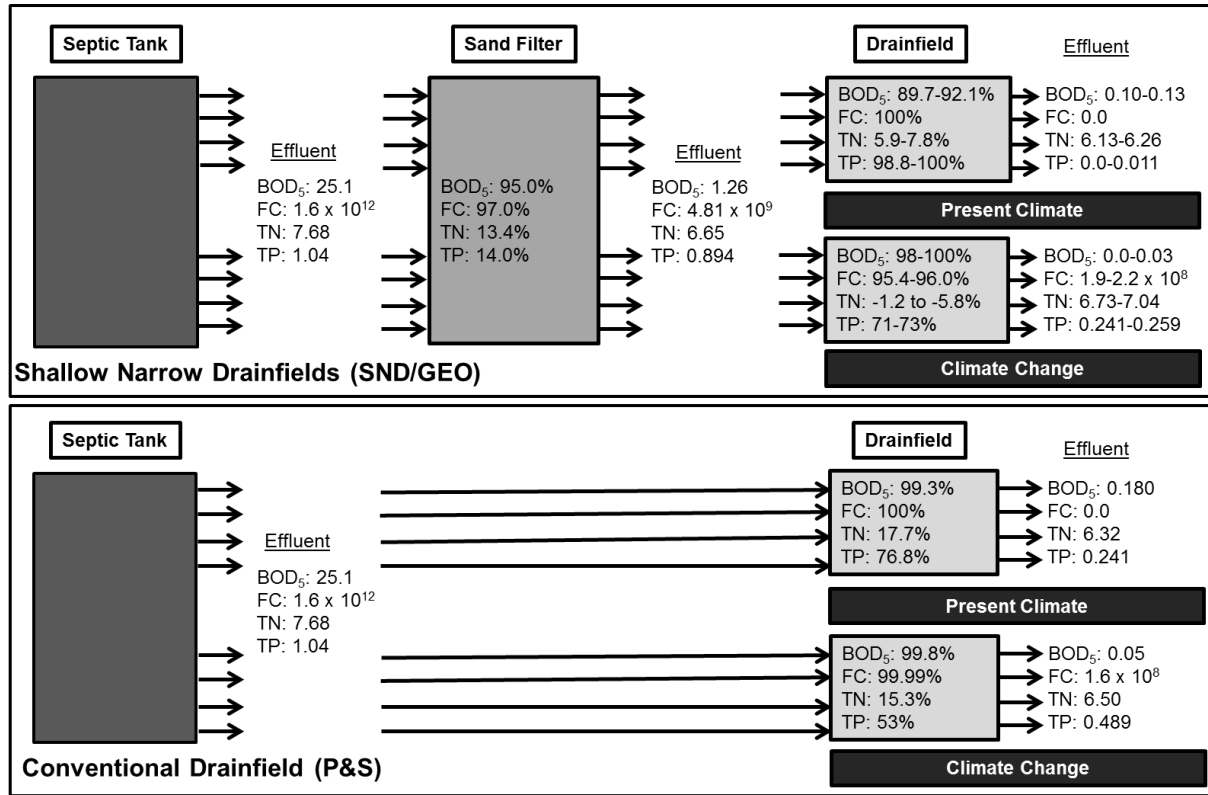

Supplement: S1 Fig — Removal values (%) are for the previous step in the treatment train. Units are kg yr-1 except for fecal coliform bacteria (FCB), which are CFU yr-1. (PDF) [file pone.0162104.s001.pdf]
