# Supplementary material for: Hell and High Water: Diminished Septic System Performance in Coastal Regions Due to Climate Change
Source: PLoS One. 2016 Sep 1;11(9):e0162104. doi: 10.1371/journal.pone.0162104 (PMC5008777; doi:10.1371/journal.pone.0162104)
Supplement: S1 File — Methodology for the propagation, addition and detection of MS2 bacteriophage in the pipe and stone (P&S), shallow narrow (SND) and Geomat® soil treatment areas (STAs). (PDF) [file pone.0162104.s002.pdf]

Supplemental Methods

Methods for MS2 bacteriophage removal experiment. ATCC® 15597-B1™ MS2 bacteriophage was propagated by addition of 0.5 mL seed to a 6 h culture of ATCC® 15597™ *Escherichia coli* strain C3000 *(E. coli)* grown in ATCC® Medium 271 at 37°C. Following cell lysis, MS2 was enumerated using to the soft agar overlay method (Adams, 1950*). MS2 was added to an aliquot of wastewater, bringing the final concentration to 8×10^7 pfu ml^{-1}. Sodium chloride was added to the virus wastewater mixtures as a conservative tracer to a final concentration of 5000 mg L^{-1} NaCl. The virus and wastewater mixtures were added to the STA in a 200 mL-dose to P&S over 1.5 h, and in a 200 mL-dose to SND and GEO in ~4.5 doses of 42 mL over 2.25 h. Output water was analyzed daily for MS2 bacteriophage plaques using the soft agar overlay method for 10 days.

* Adams M H. Bacteriophages. Interscience, New York. 1950.
