# Supplementary material for: Hell and High Water: Diminished Septic System Performance in Coastal Regions Due to Climate Change
Source: PLoS One. 2016 Sep 1;11(9):e0162104. doi: 10.1371/journal.pone.0162104 (PMC5008777; doi:10.1371/journal.pone.0162104)
Supplement: S1 Table — (PDF) [file pone.0162104.s003.pdf]

S1 Table

Parameter	SND		GEO		P&S	
	**PC**	**CC**	**PC**	**CC**	**PC**	**CC**
Length, L (cm)	102	102	102	102	56	56
Velocity, v (cm h^{-1})	1.7	1.7	1.5	1.5	0.3	0.3
Rate constant, zero order, k_0 (mg L^{-1} h^{-1})	0.047	0.0	0.05	0.021	0.033	0.044
Initial nitrate concentration, C_0 (mg L^{-1})	22-47	13-35	22-47	13-35	38-57	18-73
Damköhler number, D_a	**0.056**	**0.0**	**0.068**	**0.029**	**0.12**	**0.16**
